# Plant Virus Infection and the Ubiquitin Proteasome Machinery: Arms Race along the Endoplasmic Reticulum

**DOI:** 10.3390/v8110314

**Published:** 2016-11-19

**Authors:** Jeanmarie Verchot

**Affiliations:** Department Entomology & Plant Pathology, Oklahoma State University, Stillwater, OK 74078, USA; Verchot.lubicz@okstate.edu

**Keywords:** ubiquitin and plant viruses, UPS, Ub-proteasome pathway, RING E3 Ub-Ligase, proteasome degradation, plant immunity, CDC48, NLR receptor, endoplasmic reticulum, Argonaute, silencing suppression

## Abstract

The endoplasmic reticulum (ER) is central to plant virus replication, translation, maturation, and egress. Ubiquitin modification of ER associated cellular and viral proteins, alongside the actions of the 26S proteasome, are vital for the regulation of infection. Viruses can arrogate ER associated ubiquitination as well as cytosolic ubiquitin ligases with the purpose of directing the ubiquitin proteasome system (UPS) to new targets. Such targets include necessary modification of viral proteins which may stabilize certain complexes, or modification of Argonaute to suppress gene silencing. The UPS machinery also contributes to the regulation of effector triggered immunity pattern recognition receptor immunity. Combining the results of unrelated studies, many positive strand RNA plant viruses appear to interact with cytosolic Ub-ligases to provide novel avenues for controlling the deleterious consequences of disease. Viral interactions with the UPS serve to regulate virus infection in a manner that promotes replication and movement, but also modulates the levels of RNA accumulation to ensure successful biotrophic interactions. In other instances, the UPS plays a central role in cellular immunity. These opposing roles are made evident by contrasting studies where knockout mutations in the UPS can either hamper viruses or lead to more aggressive diseases. Understanding how viruses manipulate ER associated post-translational machineries to better manage virus–host interactions will provide new targets for crop improvement.

## 1. Introduction

The ubiquitin proteasome system (UPS) contributes to all aspects of cell biology and is widely conserved among eukaryotes. The significance of the UPS in plants is reflected by the more than 1400 genes that encode putative E3 ubiquitin (Ub)-ligases in *Arabidopsis* [1]. Many of these are involved in regulation of drought, hormone signaling, and plant immunity [2,3,4,5]. Many are well known for their interactions with microbial effectors, and few are identified for interactions with plant virus proteins [1]. Ubiquitination is a post-translational modification in which ubiquitin moieties are covalently attached to protein substrates. The process is mediated by an enzymatic cascade that begins with the E1 Ub-activating enzyme that presents an Ub molecule to the E2 Ub-conjugating enzyme. The E3 Ub-ligase transfers the Ub molecule from the E2 enzyme to the substrates, typically at Lys residues. There can be multiple Ub linkages creating elongated poly-ubiquitin chains attached to protein substrates. The outcomes of ubiquitination include proteasomal degradation of the substrate, subcellular localization or protein activation. This process of proteasomal degradation is well studied with regard to plant–pathogen interactions involved in host resistance.

There are four subfamilies of E3 Ub-ligases, which include the HECT (Homologous to E3 associated protein C-terminus), RING (really interesting new gene), U-box and CRL (Cullin–RING ligases) [6]. The most common families of E3 Ub-ligases reported to impact plant–pathogen interactions are the RING and U box E3 Ub-ligases. Both the RING E3 and U-box Ub-ligases act to transfer the Ub from an E2-Ub complex to the substrate. The majority of the functionally characterized RING and U-box E3-Ub ligases exist along the endoplasmic reticulum (ER) and contribute to ER associated degradation (ERAD), or they exist in the cytosol [6]. In *Arabidopsis*, there are approximately 469 E3 Ub-ligase genes encoding RING domain containing proteins, and very few are characterized [7]. Thus, there remains much to learn about the various biological contributions of E3 Ub-ligases.

**Figure 1:**
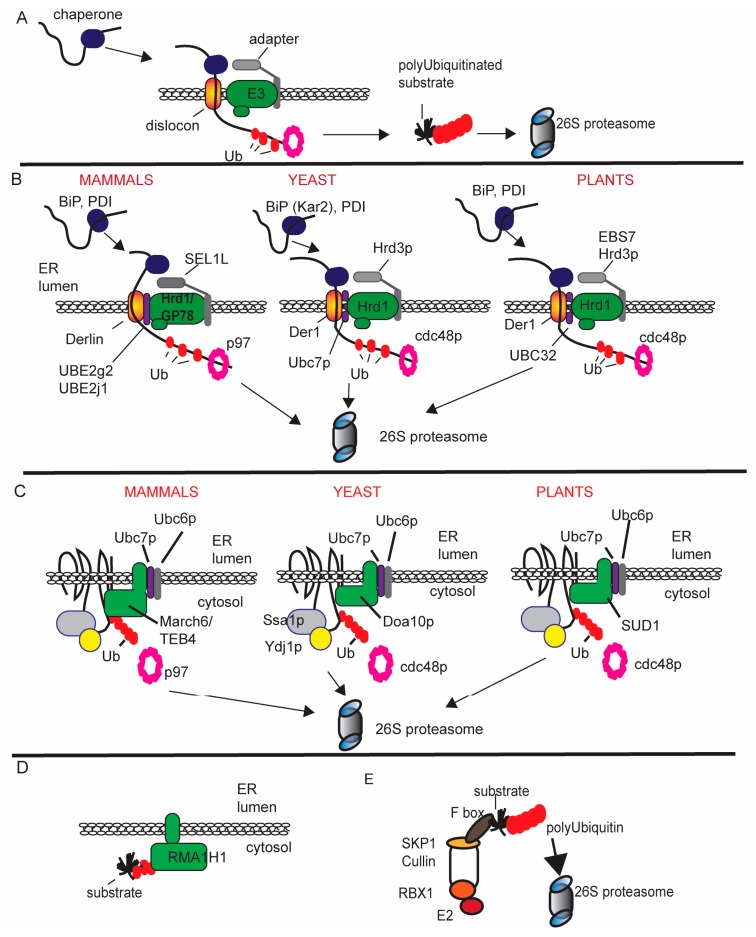
Diagrammatic representation of the classes of ubiquitin proteasome system (UPS) associated with endoplasmic reticulum (ER) associated degradation (ERAD); (**A**) shows the general features of a functional ERAD machinery and retrotranslocon found in eukaryotes; (**B**) comparison of the lumenal ERAD machinery in mammals, yeast, and plants; (**C**) comparison of the cytosolic ERAD machinery in mammals, yeast, and plants; (**D**) RMA1 class of E3 Ub-ligases that associate with the ER and modify cytosolic substrates; and (**E**) the SKP1, Cullin, F-box containing complex (SCF complex) is a cytosolic complex that is sometimes linked to ERAD. BiP: lumenal binding protein, PDI: protein disulfide isomerase; Ub: ubiquitin.

Malformed nascent proteins in the ER are identified and redirected for turnover to prevent their release to subcellular compartments where they can cause toxic damage. In this regard, ERAD essentially functions as a quality control machinery. ERAD is vital to the maintenance of healthy cells across eukaryotes [8]. The core ERAD machinery facilitates a four-step process. The first is substrate selection. The lumenal binding protein (BiP) and protein disulfide isomerase (PDI) are two of several ER resident protein foldases that identify malformed proteins for either repair or elimination. The second is retrotranslocation across the ER membrane [8,9]. The retrotranslocon provides a portal for removal of misfolded proteins from the ER (Figure 1A). Adaptor proteins at the dislocation site recruit lumenal factors that help to shuttle ERAD substrates from the folding machinery to the retrotranslocon and aid the hand-off of the substrate to the E2/E3 ubiquitin ligase complex prior to proteasomal degradation (Figure 1A). The third and fourth steps are protein ubiquitination mediated by the E1-E2-E3 cascade, and proteasome mediated degradation.

ERAD pathways are identified as ERAD-L, ERAD-M and ERAD-C based on their targets and components [10,11,12,13]. ERAD-L degrades soluble proteins in the ER that are misfolded, ERAD-M degrades membrane integrated proteins that are malformed in their transmembrane domain, and ERAD-C degrades integral membrane proteins with misfolded cytoplasmic domains. Adaptors in the ER identify substrates through the retrotranslocon and facilitate their recognition by E2/E3 Ub-ligase complex. Such adaptors include SEL1L in mammals, and Hrd3 in yeast and plants (Figure 1B) [14,15]. With respect to malformed glycoproteins, the Yos9/Os9, as well as HRd3/SEL1L, identify mannose-trimmed *N*-glycans that show faulty maturation [16,17]. EBS7 is a unique plant protein that was recently identified as an adapter that stabilizes Hrd1 (Figure 1B) [13]. HRD1/Der3p is one of the most conserved E3 Ub-ligases found in metazoans (Figure 1B). The HRD1 class of E3 Ub-ligases are multi-spanning membrane proteins. In mammals, there are two factors related to the yeast and plant Hrd1 proteins, and its namesakes are Hrd1 and gp78. Plants have HRD1a and HRD1b (Figure 1B).

In yeast, the ER bound, HRD1 functions with two E2 Ub-conjugating enzymes identified as Ubc6p and Ubc7p [18,19]. In mammals, there are four ER associated E2 Ub-conjugating enzymes known as UBE2g1, UBE2g2, UBE2j1 and UBE2j2 [20,21]. The Ubc6p has two mammalian homologues named UBE2J1 and UBE2J2 and three plant homologs are named UBC32, 33, and 34 [21]. Ubc6p and its homologues are ER-integral enzymes, whereas Ubc7p homologs are anchored to the cytoplasmic face of the ER by the integral membrane protein Cue1p [22]. The mammalian UBE2g2 coordinates with gp78 but not HRD1 for ubiquitination of some substrates, suggesting that there are special conditions governing how the E2 and E3 enzymes partner with each other [18,20]. Another conserved component of the retrotranslocon is the p97/cdc48A ATPase that acts on the cytoplasmic face of the ER and helps to pull the ubiquitinated protein from the retrotranslocon toward the proteasome (Figure 1B) [12,23].

A cytosolic ERAD pathway (ERAD-C) is maintained by another conserved E3 Ub-ligase machinery, named for the founding member in yeast Doa10p (Figure 1C). The yeast Doa10p Ub-ligase has a RING-finger domain and typically acts with Ubc6p and Ubc7p to degrade targets [18]. In yeast, Ssa1p and Ydj1p are cytoplasmic chaperones of the heat shock protein 70 (Hsp70) or Hsp40 family, which aid identification of malformed proteins for ubiquitination and degradation via Doa10p [24]. Following ubiquitination, cdc48 directs substrates to the proteasome [23,25].

## 2. Ubiquitin Proteasome System Supporting Virus Replication and Movement

In recent years, there has been mounting evidence linking protein ubiquitination machinery, the proteasome, or both to the activation and subcellular localization of virus replication or movement protein complexes [26,27,28]. The most in-depth examination of the role for the UPS in virus replication has involved studies of tomato bushy stunt virus (TBSV, genus *Tombusvirus*) and cymbidium ringspot virus (CRSV, genus *Tombusvirus*). Yeast has been used as a model genetic system to uncover critical host factors that contribute to tombusvirus infection [29,30,31], enabling the discovery of novel interactions involving cellular membranes which could be confirmed through focused experimentation in plant cells. In this yeast experimental system, cells are transformed to express the tombusviral p33 and p92 replication proteins, which function to replicate short tombusvirus replicons derived from viral defective interfering RNAs. Studies involving genome wide screens and yeast protoarrays revealed a subset of host factors associated with the viral replication machinery [32,33,34]. Homologues of many identified factors that have been identified in plants and investigations typically report parallel analysis of protein contributions to virus replication in yeast and in plants.

One major finding relevant to this discussion is that ubiquitin plays an important role in tombusvirus infection. Rad6/ubc2 and cdc34p are E2 Ub-conjugating enzymes that are components of the viral replication complexes identified in pull down assays [31,35]. Both Rad6 and cdc34p are involved in several cellular processes and, with regard to tombusvirus replication, have redundant capabilities of adding ubiquitin moieties to the tombusvirus p33 protein. Deletion of either protein impedes virus replication, showing a direct role for these factors in promoting virus infection. Normally, Rad6p contributes to DNA repair, histone ubiquitination and ERAD. Ubc2p, a plant homolog of the yeast Rad6p, is involved in flowering, salt and drought tolerance [9,36]. Rad6p/ubc2b is capable of mono-ubiquitination of most substrates in the absence of an E3 ligase, and, in the case of the tombusvirus p33 protein, data indicates that this factor attaches two ubiquitin molecules to the substrate. Both Rad6p/ubc2p and cdc34p are important for recruiting the endosomal sorting complexes required for transport (ESCRT) proteins Vps23p and Vps4p to the viral replication complex [30,31,37]. TBSV replicase forms spherule compartments along membranes, and these ESCRT proteins function in membrane bending needed to create spherular compartments. Mutational analysis indicates that p33 ubiquitination is important for virus replication.

Rsp5p is an HECT domain E3 Ub-ligase that binds to the tombusvirus p33 and p92 protein and functions along with Uba1p (E1 enzyme) and Ubc1p (E2 enzyme). Rsp5p has three domains known as C1, WW and HECT domains [29]. Deletion analysis indicates that the WW domain is involved in protein–protein interactions involving p33 and p92 and that the ubiquitin domain (HECT domain) is not important for modulating virus replication. In fact, the p92 polymerase is degraded by an endosomal and vacuolar route and not via the proteasome. Downregulation of Rsp5p leads to an increase in TBSV replication while overexpression of Rsp5p serves to downregulate TBSV replication in yeast. These data indicate that Rsp5p is a negative regulator of virus replication in yeast, but the ubiquitin ligase activity of Rsp5p is not critical for TBSV replication. Barajas et al. propose that Rsp5p may have pleiotropic effects regulating TBSV replication, making it difficult to identify one key role. However, given that p92 is supposedly degraded via an endosomal route, it is plausible to consider that Rsp5p may affect p33 and p92 recruitment of ESCRT proteins from the endosome in a manner that affects turnover of p92 [29].

Rpn11p, which is a central, stabilizing component of the 26S proteasome, is hijacked by TBSV, possibly to assist in suppression of RNA recombination in the spherular environment. Normally, Rpn11p functions to couple de-ubiquitination of substrates with proteasomal degradation, and also contributes to tombusvirus replication [31,38,39]. Rpn11p gained the attention of researchers working on TBSV replication because it was initially identified to function in peroxisome division, and TBSV replication occurs inside spherules along peroxisomal membranes. With respect to TBSV replication, Rpn11p recruits DDX3-like Ded1p DEAD box helicase proteins to replication complexes. This DEAD-box helicase is known to suppress RNA recombination during virus replication [38,40,41]. Further investigation would clarify the mechanism of recombination suppression in the spherular environment.

Other examples of virus interactions with the UPS are either less well understood or demonstrate that the virus uses the UPS to degrade preferred targets. With respect to turnip yellow mosaic virus (TYMV, genus *Tymoviruses*), the UPS judiciously degrades the 66K protein, which is the RNA dependent RNA polymerase (RdRp) component of the viral replicase machinery and is derived from a polyprotein. The outcome is lower levels of accumulating 66K protein relative to other proteins involved in the viral replication complex. Specifically, translation of the TYMV genomic RNA produces two nonstructural proteins named for their molecular weights, the 69K movement protein and the 206K polyprotein. The 206K polyprotein is proteolytically processed to produce a 66K protein and a membrane anchored 140K protein. The 140K is an intermediate polyprotein that can be further processed to the mature 98K and 42K products. The 140K polyprotein intermediate binds to the 66K polymerase and drives the complex to the chloroplast membrane where the viral replicase assembles [42]. While both the 140K and 66K proteins are derived from the same translation product, immunoblot analysis of plant extracts showed that the level of the 66K protein accumulation is lower. Through a process of phosphorylation and ubiquitination, there was a moderate level of proteasomal turnover of the 66K protein occurring during virus replication [43]. This modulates the available pools of viral RdRp and subsequently affects RNA accumulation. Modulating the components of the viral replication complex serves to reduce the potential for RNA recombination and provides temporal regulation of (−) and (+) strand RNA synthesis. In this tymovirus example, UPS serves to curb protein accumulation and consequently fine tunes the replicative processes [43].

Additional examples highlight the fact that UPS machinery degrades viral movement protein accumulation to reduce cellular toxicity caused by the build-up of foreign proteins. For example, the TYMV movement protein and the tobacco mosaic virus (TMV) coat protein can become poly-ubiquitinated during infection [44,45]. The TYMV movement protein is degraded by the proteasome. Since TMV coat protein and movement protein can form insoluble aggregates inside plant cells, researchers hypothesized that the UPS mediated the turnover of malformed insoluble coat proteins that could otherwise become toxic to the cell. Another TMV example is the movement protein (MP) that forms mobile complexes that are associated with ER and microtubules. The cell division cycle protein 48 (cdc48) participates in ERAD mechanisms and appears to deliver the viral MP to the proteasome late in infection [46]. Both examples of the TMV coat protein and MP pointed to a role of the proteasome in protecting the cell from toxic over-accumulation of TMV proteins. At the whole plant level, the 26S proteasome subunit RPN9 was shown to contribute to the systemic spread of TMV as well as the potyvirus turnip mosaic virus (TuMV). Deletions of RPN9 have pleiotropic effects on plant vascular development and auxin transport, making it difficult to discern the mechanistic interactions with TMV MP. However, data shows that deletion of RPN9 reduces virus systemic accumulation, indicating it is a positive factor in virus infection [47]. A final example is the potato virus X (PVX) TGB3 protein that has a single transmembrane domain, resides in the ER, and stimulates the unfolded protein response (UPR) and S-Phase Kinase-Associated Protein 1 (SKP1) expression. The SKP1 protein is a component of the SCF E3 Ub-ligase complex. Data shows that the TGB3 protein is turned over rapidly during virus infection [26,48,49]. Application of proteasome inhibitors increased the stable accumulation of TGB3 in virus infected cells. Silencing SKP1 directly alters the level of virus accumulation and systemic necrosis in tobacco plants. While the role of ubiquitin in this process is not established, the role of the proteasome in viral protein turnover is inferred [48,50]. These examples of TYMV, TMV and PVX demonstrate that the UPS can be subverted by some viruses to ensure cellular homeostasis and cell survival needed by both the plant and the virus to co-exist.

## 3. Hijacking the Host E3 Ub-Ligase Using Viral-Encoded F Box Proteins

The CRL are the largest category of E3 Ub-ligases, and are multi-subunit enzymes. CRLs are involved in plant hormone regulation, cell cycle regulation, development, and many other dynamic events [6]. Cullin is a subunit backbone which brings together the RING protein Ub-ligase, and a substrate recognition subunit (SRS). An adaptor protein typically links the SRS and Cullin. The SCF complex is the best studied member of the CRL category of E3 Ub-ligases and is comprised of CUL1, RBX RING protein, SKP1 adaptor, and various F-box proteins [1]. All CRLs are regulated by neddylation of the Cullin backbone, mediated by the constitutive photomorphogenesis 9 (COP9) signalosome. Arabidopsis has more than 700 F-box proteins and the breadth of substrates that are identified by these factors are very much unknown. There are many examples of F-box proteins contributing to phytohormone signaling including auxin, jasmonate, giberillin, and ethylene signaling [51,52,53,54,55]. One example is the F-box protein, Transport Inhibitor Response 1 (TIR1), which binds to auxin and functions to degrade auxin proteins. These auxin proteins are repressors of auxin activity by binding to and repressing auxin responsive transcription factors. Knockdown of certain F-box proteins such as TIR1, Arabdillo-1 or -2, demonstrated that these proteins play a critical role in lateral root formation. In this regard, the SCF complex serves to degrade negative regulators of hormone responses. 

**Figure 2:**
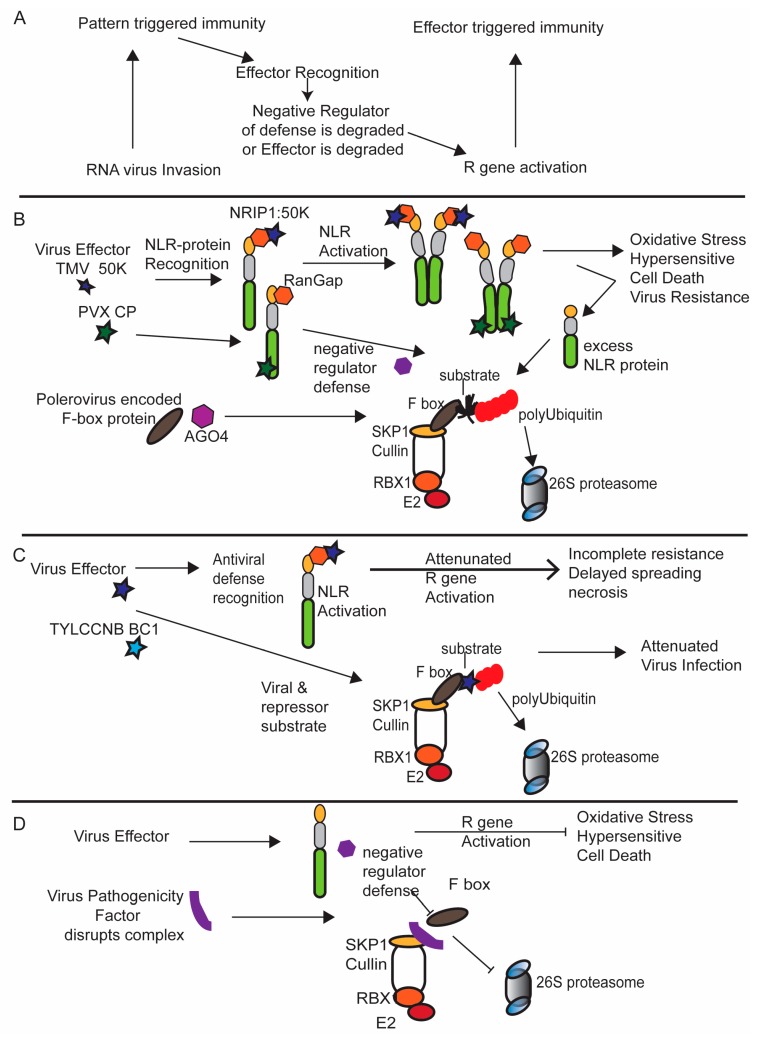
Diagrammatic representation of viral protein interactions with the UPS with respect to the molecular arms race; (**A**) zig-zag model demonstrating the co-evolution of viruses with pathogen associated molecular patterns (PAMP)-triggered immunity (PTI) and effector-triggered immunity (ETI); (**B**) two examples of tobacco mosaic virus (TMV) and potato virus X (PVX) effectors that activate N-protein or Rx-protein (NLR proteins) leading to activation of ETI and a hypersensitive cell death. With respect to N-protein mediated resistance, nuclear receptor interaction protein (NRIP) is a co-factor that binds the viral 50K effector. The co-factor for Rx is Ran–Gap. Activation of the respective NLR proteins involves effector recognition, followed by dimerization or oligomerization. This is followed by oxidative stress, hypersensitive response and virus resistance. This model suggests that unknown factors that may serve as a negative regulator of the NLR protein, which blocks autoactivation, may be relayed to the SCF complex for ubiquitination and 26S proteasome degradation. In which case, the SCF complex is central to regulating NLR protein activation. Another method for protecting the cell from auto-activation involves routine turnover of NLR proteins that may be excessive or have completed the necessary activation of defense responses. With regard to PTI, poleroviruses encode an F-box protein that binds to Argonaute 4 (AGO4), relaying it to the SCF complex for ubiquitination and degradation. This serves to compromise PTI; (**C**) a proposed model for degradation of viral effectors by the SCF complex or ERAD machinery to evade immune recognition; and (**D**) certain viruses are known to interact with S-Phase Kinase-Associated Protein 1 (SKP1) but do not appear to function as F-box proteins. One possibility is that these proteins insert into the SCF complex and disrupt its ability to function in the degradation of negative regulators of defense as in panel A. In this scenario, the immune system may be blocked.

A handful of plant viruses are known to encode proteins that interact with F-box proteins, encode their own F-box proteins or interact with SKP1. In the case of Beet necrotic yellow vein virus (BNYVV; a Benyvirus), the P25 proteins are identified as a pathogenicity factor responsible for rhizomania symptoms, also known as “crazy root” symptoms [56]. The classic symptoms of rhizomania are expansive numbers of secondary roots that give the main taproot a bearded appearance. The Gilmer laboratory transgenically expressed the BNYVV P25 protein in *Arabidopsis* and plants showed more lateral roots than the non-transgenic controls, had higher levels of auxin, and were more responsive to exogenous applications of 2,4-Dichlorophenoxyacetic acid (2,4-D; an auxin analog) [57]. Three genes encoding F-box proteins were upregulated in these transgenic *Arabidopsis* plants. One F-box protein named the AtFBK protein is a homolog of the sugar beet FBK protein, which binds to the P25 protein, but this interaction does not involve the substrate binding domain. The FBK protein also interacts with SKP1, ascribing this F box protein to the SCF complex. In *Arabidopsis*, there are two homologues of SKP1 known as ASK1 and ASK2. Competitor studies suggest that P25 interferes with FBK and ASK1 interactions but not FBK and ASK2 interactions. These data suggest that P25 directs FBK to a specific adaptor protein and monitors SCF complex formation. The data argue that P25 targets the F-box protein to downregulate cellular necrosis while promoting rhizomania symptoms [58]. If we apply a model in which there is a hidden *R* gene, it is also possible that the P25-F box interaction eliminates the ability of the SCF complex to function in de-repressing *R* gene function (Figure 2). Such a model is supported by experiments where the same F-box protein was overexpressed and the result was activation of programmed cell death. Such a model suggests that the viral factors disable the incorporation of certain subunits into the SCF machinery in favor of others, perhaps to redirect the complex toward monitoring phytohormone signaling and away from immune recognition.

Poleroviruses interestingly encode the P0 protein that combines the F-box protein interactions to target degradation of the host gene silencing machinery. The polerovirus P0 protein can be incorporated into the SCF complex and identifies substrate Argonaute 1 (AGO1) for degradation. AGO1 is central to the RNA-induced silencing complexes (RISC), which incorporates anti-viral small interfering RNAs (siRNAs) as part of the gene silencing machinery. The polerovirus P0 protein is a silencing suppressor that enables turnover of AGO1 to limit anti-viral defenses [59,60]. Another viral encoded F box protein is the rice black-streaked dwarf virus (RBSDV; genus *Fijivirus*) P7-2 protein, which is likely incorporated into cellular SCF complexes. P7-2 has a Mr of 36 kDA and interacts with the ZmSKP1 protein [61]. Unlike the polerovirus F-box proteins, the P7-2 protein is not a gene silencing suppressor and does not target AGO for degradation. The substrate identified by P7-2 is not known. One possibility is that the P7-2 protein is incorporated into the SCF complex to degrade other virus selected cellular substrates. Another possibility is that the P7-2 protein disrupts the SCF complex to dismantle cellular defenses that are involved in pathogen associated molecular patterns (PAMP)-triggered immunity (PTI) or effector-triggered immunity (ETI) [61] (Figure 2D).

## 4. The Ubiquitin Ligase 26S Proteasome System and the Molecular Arms Race

In the evolution of plant–pathogen interactions, plants have developed several surveillance systems to recognize the invaders and activate defenses to arrest pathogen infection [62]. A detection system that identifies PAMPs and activates immune responses is known as PTI. Pathogens that are adapted to their hosts are capable of inhibiting or circumventing PTI (Figure 2). PTI involves cell surface receptor-like kinases (RLKs), a class of proteins that play crucial roles in immunity, cell differentiation, and plant growth [63]. With advancement of the molecular arms race, plants have evolved classic resistance (R) proteins to detect effectors and trigger disease resistance known as effector-triggered immunity (ETI) [63,64,65]. ETI is often accompanied by a hypersensitive response (HR; Figure 2A). PTI and ETI employ different immune receptors but use similar signaling networks to arrest the pathogen and defend the host. 

Plant viruses deposited by vectors into the cytoplasm are not known to interact with cell surface receptors. Virus infections are typically combatted intracellularly by the RNA silencing machinery as the main form of PTI (Figure 2A). Virus derived small RNAs (viRNAs) from positive strand RNA viruses are produced from the long double strand RNA replication intermediates by an enzyme called Dicer like 4 (DCL4). One strand of the viRNAs are assembled into RISCs, which contains central small RNA binding protein known as Argonaute (AGO). These RISC complexes are directed by the guiding viRNA to base-pairs with homologous viral genomic strands and continue the cycle of RNA degradation. In this arms race, viruses have evolved suppressors or RNA silencing that can attack the RNA silencing machinery to stave off the anti-viral RNA defense [66,67]. This can involve degradation of components of the RNA machinery by the 26S proteasome. For example, the poleroviruses encoded P0 protein, which is also an F-box protein, is a silencing suppressor that blocks the function of the RISC complex [59,60,68,69]. The F-box domain of the P0 protein interacts with the SKP1 component of the SCF complex (Figure 2B). Several studies revealed that the P0 protein of Cucurbit aphid-borne yellows virus (CABYV), Beet western yellows virus (BWYV), and Sugarcane yellow leaf virus (SYLV) recognizes AGO proteins and targets them for degradation [59,60,68,69,70,71]. P0-mediated degradation of AGO disrupts the antiviral gene silencing functions to promote infection. More recently, the potyvirus viral silencing suppressor protein named HC-Pro was demonstrated to interact with subunits of the host proteasome. The potato virus Y, lettuce mosaic virus, and papaya ringspot virus HC-Pro proteins modulate the activities of the 20S proteasomal subunits PAA, PBB, and PBE [63,72,73,74,75]. The 20S proteasome also contains RNase activity, and so it is not clear if these interactions inhibit or RNase activity as well as protein degradation. However, these interactions do appear to impact virus accumulation.

With respect to ETI, the role for the SCF complex in the arms race between pathogen and host is exemplified in studies involving TMV and *N*-gene mediated virus resistance in tobacco or PVX and Rx mediated resistance in potato (Figure 2B). The *N*-gene recognizes the helicase domain of the TMV replicase while Rx recognizes the PVX coat protein (CP). The *N*-gene belongs to one class of toll-interleukin 1-receptor (TIR) nucleotide-binding site leucine-rich repeat (NB-LRR) genes [76] and Rx belongs to a class of coil–coil (CC) NB-LRR genes (NLR). The N-protein mediated resistance requires the cofactors Rar1 and EDS1, which function upstream of oxidative stress signaling and programmed cell death machineries. In a quiescent state, the N and Rx protein adopts a folded inactive form. Recognition of the viral effectors TMV 50K or PVX coat protein (CP) alters the NLR protein conformation and enables these proteins to oligomerize (Figure 2B) [77]. During N-mediated resistance, Rar1 associates with SGT1, which, in turn, binds to the SKP1 component of the SCF complex. Both Rar1 and SGT1 are required for N-mediated resistance to TMV. A model was proposed by Liu et al. (2002) in which the N-protein uses Rar1 and SGT1 to relay protein substrates to the SCF complex for ubiquitination and degradation. The identities of the protein substrates are not known but are likely to be negative regulators of defense (Figure 2B) [76,78]. In the case of Rx and several other NLR proteins, overexpression can lead to auto activation of immune responses. The SCF complex and 26S proteasome play a central role in preventing auto-activation by over accumulation of NLR proteins (Figure 2B). In fact, a recent study identified Cdc48A as a negative regulator of NLR accumulation and autoimmunity [25]. However, there is not sufficient information to know whether the ERAD machinery is also responsible for regulating NLR protein accumulation.

Beyond virus control through PTI related RLKs or ETI related NLR proteins, geminiviruses, which have single strand circular DNA genomes and replicate in the nucleus, provide an example of how a certain class of viruses can use the SCF-mediated ubiquitin system to elude host defenses. This goes beyond the poleroviruses or fijivirus encoded F-box proteins that can be incorporated into SCF complexes [69,71]. To better elaborate this scenario, the geminivirus C2 is a multifunctional protein that serves as a transcriptional activator protein, a repressor of host defenses, and suppressor of gene silencing. The C2 protein of Tomato yellow leaf curl virus and Beet curly top virus interacts with the CSN5 complex, which functions to remove ubiquitin-like moieties from the neddylated Cullin. Viral proteins that hinder Cullin modification also hinder the function of plant SCF complexes. The action of the C2 protein causes several defects and appears to inhibit jasmonate defense signaling machinery [79,80]. The exact outcomes of a malfunctioning SCF complexes in relation to plant virus infection are not known at the mechanistic level. However, it is easy to imagine that cellular repressors of defense are blocked from degradation, and this could suppress host defenses favoring virus infection (Figure 2B).

The UPS is also central to the arms race involving geminiviruses because, in addition to the example of C2 disruption of the SCF complex, the UPS can also play a central role in anti-viral mechanisms (Figure 2C). Specifically, tobacco and tomato encode RFP1, which attacks tomato yellow leaf curl China virus (TYLCCV) and the virus-associated betasatellite (TYLCCNB). RFP1 expressing plants show reduced symptoms as the result of ßC1 protein degradation by the ubiquitin/26S proteasome machinery [81,82]. In tomato and tobacco, ubc3, an E2 Ub-conjugating enzyme and RFP1 coordinate the ubiquitin modification of the viral ßC1 protein, tagging it for degradation by the 26S proteasome. Thus, RFP1 and ubc3 coordinately provide an antiviral mechanism that directly targets viral proteins for degradation in a manner that directly attenuates virus infection (Figure 2C).

## 5. Conclusion

The UPS machinery contributes to adaptive cellular responses to environmental stressors, change in ER conditions, and virus infection. Given the plethora of genes that are categorically ascribed to the UPS, we are still uncovering the various contributions of this system to plant cell biology. However, there are some notable themes emerging that indicate that the UPS is central to plant virus infection. One theme that stands out is the role of ubiquitin machinery supporting tombusvirus and tymovirus replication. With respect to tombusvirus replication, Rad6/ubc2, cdc34p and Rsp5p provide three very interesting examples where separate E2, E3, and proteasome components are incorporated into the viral replication machinery to support interactions with a subset of ESCRT proteins needed to shape a membrane environment along the peroxisome needed to support virus replication. In addition, at least one component of the proteasome is known to be highjacked by the virus, possibly to assist in suppression of RNA recombination in the spherular environment. These data highlight the controlling role that the UPS machinery plays in virus infection making individual component factors useful targets for crop improvement. The application of these discoveries is to identify the best component factors where introducing a mutation into the gene could suppress virus infection and with perhaps limited impact on crop yield. Given the new technologies for transformation, this becomes a reasonable strategy for transferring this knowledge to crop improvement. The tombusvirus example, in fact, highlights the value of in-depth studying of the molecular mechanisms of plant virus infection for identifying valuable targets for crop improvement while understanding how these targets function.

With respect to the role of UPS in virus intercellular and systemic movement, studies of TMV and PVX suggest that the UPS machinery is required for rapid turnover of viral proteins to reduce membrane damage and cellular toxicity. The UPS machinery also contributes to antiviral immunity either by monitoring negative repressors of NLR activation, degrading viral effectors, or degrading AGO to suppress gene silencing.

With respect to ERAD, the ERAD-C, -L, and -M pathways for protein degradation are well described in mammals and yeast, but far less is known in plants. Data thus far in plants confirm that the HRD1 pathway regulates misfolded proteins in the ER lumen and intramembrane domains, while the SUD1/Doa10/TEB4 pathway regulates malformed proteins in the cytosol. Until now, research into the topic of plant virus related protein ubiquitination and degradation has not uncovered a role for ER resident HRD1 or SUD1. The generally limited information surrounding the nature of the ERAD machinery in plants also limits our understanding of its role in plant virus infection. It is worth noting that most of the research to uncover ERAD machinery in plants has employed abiotic stressors, microbial effectors, and hormone applications. Similar research carried out using plant viruses as stressors are likely to uncover novel aspects of the machinery that could propel our understanding of cellular protein degradation machinery and ERAD substrates in new directions.

The SCF complex is the best studied of the E3 Ub-ligases in plants, and is thus far identified in the turnover of AGO1, NLR gene mediated anti-virus innate immunity as well as the direct regulation of plant virus infection. The models presented thus far indicate that ubiquitin is important for intervention during host defense responses to virus infection and can be used to compromise the host gene silencing machinery. A combination of genetic and biochemical approaches will be needed to provide new understanding of the mechanisms that are exploited by viruses to achieve successful biotrophic infections.

The ER is central to plant virus replication, translation, maturation, and egress. ER resident chaperones can be hijacked from the ER lumen or cytosolic side of the ER and incorporated into viral replication complexes to promote viral RNA accumulation. Ubiquitin modification of ER associated cellular and viral proteins, alongside the actions of the 26S proteasome are vital for the regulation of infection. ER associated ubiquitination as well as cytosolic ubiquitin ligases can be targeted by viruses as a means to direct the ubiquitin proteasome system (UPS) to new targets. Such targets include necessary modification of viral proteins which may stabilize certain complexes, or modification of AGO to suppress gene silencing. Combining the results of unrelated studies, many positive strand RNA plant viruses, as well as the single strand DNA geminiviruses, appear to interact with E3 Ub-ligases to provide novel avenues for controlling the deleterious consequences of disease. Viral interactions with the UPS serves to regulate virus infection in a manner that promotes replication and movement, but also modulates the levels of RNA accumulation to ensure successful biotrophic interactions. In some instances, knockout mutations in the UPS can hamper virus infection. In other instances, knockout mutations lead to higher levels of virus accumulation, more aggressive disease, and/or cell death. Understanding how viruses manipulate ER associated post-translational machineries to better manage virus–host interactions will provide new targets for crop improvement.
